# Adapting Attachment and Biobehavioral Catch‐up for infants and young children with intellectual disabilities and developmental delays in South Africa: Reflections and recommendations from local stakeholders

**DOI:** 10.1002/imhj.22027

**Published:** 2022-12-20

**Authors:** Ahmed Riaz Mohamed, Paula Sterkenburg, Esmé van Rensburg, Carlo Schuengel

**Affiliations:** ^1^ Department of Clinical Child and Family Studies and Amsterdam Public Health Research Institute Vrije Universiteit Amsterdam Netherlands; ^2^ Department of Psychology North‐West University Potchefstroom South Africa; ^3^ Department of Assessment and Treatment Bartiméus Doorn Netherlands; ^4^ Department of Psychology University of Pretoria Pretoria South Africa

**Keywords:** Attachment and Biobehavioral Catch‐up, developmental delays, intellectual disability, sensitive parenting, South Africa, Intervención de Afectividad y Alcance del Bio‐comportamiento, discapacidad intelectual, retrasos en el Desarrollo, sensibilidad en la crianza, Sudáfrica, Attachement et rattrapage bio‐comportemental (ABC), Déficience intellectuelle, Retard du développement, Parentage sensible, Afrique du Sud, Attachment and Biobehavioral Catch‐up, geistige Behinderung, Entwicklungsverzögerungen, sensible Elternschaft, Südafrika, 愛着と生物行動学的キャッチアップ、知的障がい、発達遅延、敏感な育児、南アフリカ, 关键词:依恋和生物行为追赶, 智力残疾, 发育迟缓, 敏感性育儿, 南非, **الكلمات المفتاحية**: التعلق واللحاق السلوكي الحيوي ، الإعاقة الذهنية ، التأخر في النمو ، الرعاية الوالدية الحساسة ، جنوب إفريقيا

## Abstract

Attachment and Biobehavioral Catch‐up (ABC) is an intervention targeted at enhancing the socioemotional and regulatory functioning of at‐risk infants. However, to use the ABC for infants/toddlers with intellectual disabilities/developmental delays (ID/DD) and in novel cultural contexts, such as South Africa, adaptations may be required. This study aimed, therefore, to explore the opinions of clinical experts and perceptions of caregivers regarding the use of ABC for children with ID/DD in South Africa. It also sought to incorporate the experiences of families of children with ID/DD who received, and intervenors who delivered, ABC in its first implementation in South Africa. Semi‐structured interviews were conducted with 18 participants. Thematic analysis explicated 12 main themes: Intervention Strengths, Points of Concern, and Recommendations (Experts); Focus on Caregiver‐child Relationship, and Intervention Targets and Duration (Caregivers); Benefits of ABC, and Cultural Considerations (ABC Recipients); Focused and Targeted, Value of Feedback, Supportive Supervision, Working Alliance, and Challenges Experienced (ABC Intervenors). Practice and training recommendations include psychoeducation for parents and training for intervenors that is ID/DD‐specific, expanding supervision capacity, building intervenors’ cultural/linguistic sensitivity and competence, accessing referral networks, including local Community Health Workers as intervenors, and greater flexibility in how the sessions are organized.

## Resumen

La intervención de Afectividad y Alcance del Bio‐comportamiento (ABC) está dirigida a mejorar el funcionamiento socioemocional y regulatorio de los infantes bajo riesgo. Sin embargo, para usar el ABC para infantes y niños pequeñitos con discapacidades intelectuales / retrasos en el desarrollo (ID/DD) y dentro de nuevos contextos culturales, tal como en Sudáfrica, se requieren adaptaciones. Este estudio se propuso, por tanto, explorar las opiniones de expertos clínicos y las percepciones de quienes prestan el cuidado acerca del uso del ABC con niños con ID/DD en Sudáfrica. También buscaba incorporar las experiencias de familias de niños con ID/DD que recibían, así como de practicantes de la intervención que ofrecían el servicio de ABC durante su primera implementación en Sudáfrica. Se llevaron a cabo entrevistas semiestructuradas con 18 participantes. Análisis temáticos explicaron los 12 principales temas: Puntos Fuertes de la Intervención, Puntos que Preocupan y Recomendaciones (Expertos); Enfoque en la Relación Cuidador‐Niño y Metas de Enfoque y Duración de la Intervención (Cuidadores); Beneficios del ABC y Consideraciones Culturales (Quienes recibían el ABC); Lo Enfocado y Diseñado como Meta, Valor de Ideas Recibidas, Supervisión con Apoyo, Alianza de Trabajo y Retos Experimentados (Practicantes de la Intervención ABC). Las recomendaciones de práctica y entrenamiento incluyen la psico‐educación de padres, entrenamiento a quienes practican la intervención específicamente orientada a ID/DD, expandir la capacidad de supervisión, formar la sensibilidad y competencia cultural/lingüística de quienes practican la intervención, acceso a los sistemas de referencias, incluir Trabajadores de Salud Comunitarios como practicantes de la intervención y mayor flexibilidad en cómo organizar las sesiones.

## Résumé

L'attachement et le rattrapage bio‐comportemental (en anglais Attachment and Biobehavioral Catch‐up, soit ABC) est une intervention ciblée sur l'amélioration du fonctionnement socio‐émotionnel et régulatoire de bébés à risques. Cependant, pour utiliser l'ABC pour des bébés/jeunes enfants avec des retards intellectuels et des retards du comportement (ID/DD) et dans des contextes culturels nouveaux, comme l'Afrique du Sud, des adaptations pourraient être nécessaires. Par conséquent cette étude s'est donné pour but d'explorer les opinions d'experts cliniques et les perceptions des personnes prenant soin d'un bébé quant à l'utilisation de l'ABC pour des enfants avec ID/DD en Afrique du Sud. On a aussi cherché à incorporer les expériences de familles d'enfants avec ID/DD qui ont reçu l'ABC, ainsi que des intervenants qui l'ont facilité, dans sa première mise en oeuvre en Afrique du Sud. Des entretiens semi‐structurés ont été faits avec 18 participants. Une analyse thématique a explicité 12 thèmes principaux: Forces de l'Intervention, Points d'Inquiétude, et Recommandations (Experts); Accent sur la Relation Personne prenant soin de l'enfant‐enfant et Cibles d'Intervention et Durée d'Intervention (Personnes prenant soin d'un bébé); Bénéfices de l'ABS, et Considérations Culturels (personnes ayant reçu l'ABC); Ciblé et Centré, Valeur du Retour, Supervision de Soutien, Alliance entre entités, et Défis Vécus (Intervenants ABC). Les recommandations de pratique et de formation incluent une psychoéducation pour les parents, une formation pour les intervenants qui est spécifique à l'ID/DD, l’élargissement de la capacité de supervision, le développement de la sensibilité et de la compétence culturelle/linguistique des intervenants, l'accès aux réseau d'orientation, y compris des Travailleurs de Santé Communautaires en tant qu'intervenants, et une plus grande flexibilité dans la manière dont les sessions sont organisées.

## Zusammenfassung

Die Anpassung des Programms „Attachment and Biobehavioral Catch‐up“ an Säuglinge und Kleinkinder mit geistigen Behinderungen und Entwicklungsverzögerungen in Südafrika: Überlegungen und Empfehlungen von lokalen Akteur:innen

„Attachment and Biobehavioral Catch‐up; ABC) ist eine Intervention, die darauf abzielt, die sozio‐emotionalen und regulatorischen Funktionen von gefährdeten Säuglingen zu verbessern. Wenn der ABC bei Säuglingen/Kleinkindern mit geistigen Behinderungen/ Entwicklungsverzögerungen (disabilities/developmental delays; ID/DD) und in neuen kulturellen Kontexten ‐ wie z. B. in Südafrika ‐ angewendet wird, können jedoch Anpassungen erforderlich sein. Ziel dieser Studie war es daher, die Meinung von klinischen Expert:innen und die Wahrnehmung von Betreuungspersonen in Bezug auf die Anwendung des ABC für Kinder mit ID/DD in Südafrika zu untersuchen. Außerdem sollten die Erfahrungen von Familien mit Kindern mit ID/DD, die bei seiner Ersteinführung in Südafrika an ABC teilgenommen hatten, und diejenigen der durchführenden Interventions‐Fachkräfte einbezogen werden. Mit 18 Teilnehmenden wurden halbstrukturierte Interviews geführt. Aus einer thematischen Analyse ergaben sich 12 Hauptthemen: Stärken der Intervention, Bedenken und Empfehlungen (Expert:innen); Fokus auf die Beziehung zwischen Betreuungsperson und Kind sowie Ziele und Dauer der Intervention (Betreuungspersonen); Vorteile des ABC und kulturelle Überlegungen (ABC‐Teilnehmende); Fokussiert‐ und Zielgerichtetheit, Wert des Feedbacks, unterstützende Supervision, Arbeitsbündnis und erlebte Herausforderungen (ABC‐Interventions‐Fachkräfte). Zu den Empfehlungen für Praxis und Ausbildung gehören Psychoedukation für die Eltern und eine ID/DD‐spezifische Ausbildung für die Interventions‐Fachkräfte, eine Erweiterung der Supervisionskapazitäten, Förderung der kulturellen/sprachlichen Sensibilität und Kompetenz der Interventions‐Fachkräfte, Zugang zu Überweisungsnetzwerken, Einbeziehung lokaler „Community Health“‐Kräfte als Interventions‐Fachkräfte und eine größere Flexibilität bei der Organisation der Sitzungen.

## 抄録

南アフリカにおける知的障がいおよび発達遅延のある乳幼児に対する愛着と生物行動学的キャッチアップの適応:現地の関係者の考察と提言

愛着と生物行動学的キャッチアップ (ABC) は、リスクのある乳幼児の社会情緒的・調節的機能を高めることを目的とした介入である。しかし、知的障がい・発達遅延 (ID/DD) のある乳幼児や南アフリカのような新しい文化的背景でABCを使用するためには、‐適応させることが必要だろう。そこで本研究では、南アフリカのID/DD児に対するABCの使用について、臨床専門家の意見と養育者の認識を調査することを目的とした。また、南アフリカで初めてABCを実施したID/DDの子どもの家族と、ABCを実施した介入者の経験も取り入れることを目指した。18名の参加者に対して半構造化面接を実施した。主題分析により、12の主要なテーマが明らかにされた。介入の長所、懸念点、推奨事項 (専門家) 、養育者と子どもの関係性への焦点、介入目標と期間 (養育者) 、ABCの利点、文化的配慮 (ABC受益者) 、焦点と目標、フィードバックの価値、支持的スーパービジョン、作業同盟、経験された困難 (ABC介入者) 。実践と訓練に関する推奨事項には、親に対する心理教育、介入者に対するID/DDに特化した訓練、スーパービジョン能力の向上、介入者の文化的/言語的感受性と能力の構築、地域のコミュニティヘルスワーカーを介入者に加えた紹介ネットワークへのアクセス、セッションの構成方法における柔軟性の向上が含まれている。

## 摘要

“依恋和生物行为追赶” (ABC) 是一种旨在增强高危婴儿社交情感和调节功能的干预措施。然而, 在南非等新的文化背景下, 将ABC用于智力残疾或发育迟缓 (ID/DD) 的婴幼儿, 可能需要进行调整适应。因此, 本研究旨在探讨临床专家的意见和护理人员对南非ID/DD儿童使用ABC的看法。研究还试图将接受ABC的ID/DD儿童家庭以及实施ABC的干预者的经验融入南非首次开展ABC的实践中。对18名参与者进行了半结构化访谈。主题分析阐述了12个主题:干预优势、关注点和建议 (专家) ;关注护理人员与儿童的关系、干预目标和持续时间 (护理人员) ;ABC的好处和文化考量 (ABC受众) ;重点突出、目标明确、反馈价值、支持性监督、工作联盟和经历的挑战 (ABC干预者) 。实践和培训建议包括对父母进行心理教育和针对ID/DD的干预者培训, 扩大监督能力, 培养干预者的文化、语言敏感性和能力, 使用转诊网络, 将当地“社区卫生工作者”纳入干预者, 以及如何更加灵活地组织课程。

## ملخص

برنامج التعلق واللحاق السلوكي الحيوي (ABC) هو تدخل يستهدف تعزيز الأداء الاجتماعي والعاطفي والتنظيمي للرضع المعرضين للخطر. ومع ذلك ، فإنه يحتاج إلى تكييف وتعديل حتى يمكن استخدامه للرضع / الأطفال الصغار الذين يعانون من إعاقات ذهنية / تأخيرات في النمو وفي السياقات الثقافية الجديدة ، مثل جنوب إفريقيا. تهدف هذه الدراسة إلى استكشاف آراء الخبراء الاكلينيكيين وتصورات مقدمي الرعاية فيما يتعلق باستخدام ABC للأطفال الذين يعانون من إعاقات ذهنية أو تأخر نمو (DD‐ID) في جنوب إفريقيا. كما سعت إلى دمج تجارب أسر الأطفال والمختصين الذين تعاملوا مع برنامج (ABC) في أول تطبيق له في جنوب إفريقيا. أجريت مقابلات شبه منظمة مع 18 مشاركا. أسفر التحليل الموضوعي عن 12 موضوعًا رئيسيًا: جوانب القوة في التدخل ، جوانب تدعو للقلق ، والتوصيات (الخبراء) ؛ التركيز على العلاقة بين مقدم الرعاية والطفل ، وأهداف التدخل ومدته (مقدمو الرعاية) ؛ فوائد ABC والاعتبارات الثقافية (متلقو ABC) ؛ التركيز والاستهداف ، قيمة التعليقات ، الإشراف الداعم ، تحالف العمل ، والتحديات التي تم مواجهتها. (متدخلو ABC). تتضمن توصيات الممارسة والتدريب التثقيف النفسي للآباء وتدريب المتدخلين في الإعاقة الذهنية وتأخر النمو ، وتوسيع القدرة على الإشراف ، وبناء الحساسية والكفاءة الثقافية / اللغوية للمتدخلين ، والوصول إلى شبكات الإحالة ، بما في ذلك العاملين في الصحة في المجتمع المحلي كمتدخلين ، ومرونة أكبر في كيفية تنظيم الجلسات.

## 1 INTRODUCTION

Attachment and Biobehavioral Catch‐up (ABC) is an attachment‐based intervention aimed at promoting attachment through enhancing parenting practices by facilitating sensitive and responsive caregiving for at‐risk families. Randomized controlled trials have demonstrated the effect of ABC on improving parenting outcomes including, for example, caregiver sensitivity (e.g., Yarger et al., 2020), as well as child outcomes related to attachment (e.g., Zajac et al., 2020) and cortisol regulation (e.g., Berlin et al., 2019). Thus, there is an established, and growing, body of evidence, across a diverse range of outcomes for infants and toddlers, and their (foster) parents, supporting the effectiveness of ABC in contributing to various facets of child development in both laboratory and community‐based settings.

During 10 home visits, in‐the‐moment feedback on real‐time parent‐child interactions is used as the primary mechanism to scaffold shifts in parenting behaviors in relation to the ABC's central themes. Addressed first are parents’ responses to their child's bids for nurturing. Second, greater synchrony is promoted by encouraging parents to follow their child's lead. Third, the intervention assists parents to avoid intrusive and/or frightening parenting behaviors. In addition, ‘voices from the past’ are addressed, drawing attention to how parents’ own attachment experiences may influence their parenting behaviors. In closing, the gains made during the process are practiced and further consolidated (see Dozier and Bernard (2019) for a more detailed description of the intervention process).

Delays in a child's development are thought to impact parental functioning significantly (Herring et al., 2006). Hence, the ABC's three focal tenets—nurturing, following the lead, and reducing intrusive behaviors—could be especially useful for parents of children with ID/DD who may have difficulty understanding and interpreting their child's signals, and may struggle to notice the child's need for nurturing (Oppenheim et al., 2007). Furthermore, due to the child's developmental lag, parents may feel the need to stimulate learning by turning interactions into an opportunity for teaching thus taking the lead rather than following the child's lead (Dozier & Bernard, 2019). Finally, interactions between parents and their children with ID/DD are often characterized by parenting that is more intrusive (Brown et al., 2011; Floyd et al., 2004), suggesting the ABC's focus on reducing intrusive and frightening behaviors could be beneficial.

While support for parents of children with ID/DD is available in various forms through collectives and organizations in South Africa such as, for example, the Western Cape Forum for Intellectual Disability, the Chaeli Campaign, Shonaquip (Trafford et al., 2021), and the Disabled Children's Action Group (Philpott & Muthukrishna, 2019), none of these organizations adopt a parenting‐focused approach based on principles rooted in developmental psychology and attachment theory, which is where the ABC focusses its attention. This focus is rooted in the idea that children with ID/DD can, and do, develop attachment relationships much like typically developing children but that this may take a different course and, therefore, parents and caregivers may need additional support to empower them in their caregiving capacities (Vandesande et al., 2019a). This is to enhance responsive caregiving, a necessary component of nurturing care, which is essential for children to reach their developmental potential (Richter, 2018). This is likely to make a contribution to inclusive early childhood development, and ultimately, greater participation in society for children with ID/DD (Wertlieb, 2018).

**Key Findings**

1. ABC was perceived and experienced as beneficial and relevant for children with ID/DD in South Africa.
2. Integrating local cultural conceptualisations, community‐based intervenors and access to adjunct psychosocial support can further facilitate the ABC's applicability in South Africa.
3. ID/DD‐themed psychoeducation to address caregiver expectations, adapting activities to the child's capabilities and training intervenors in identifying idiosyncratic signal expressions of children with ID/DD can enhance the ABC's application in this cohort.

The ABC may, therefore, have the potential to offset the psychosocial risks faced by infants and young children diagnosed with ID/DD. Although individual studies have found an increased risk of insecure and disorganized attachments for children with ID (Feniger‐Schaal & Joels, 2018; Ganiban et al., 2000), recent reviews have concluded that the evidence for this risk is inconclusive (Hamadi & Fletcher, 2021; Potter‐Dickey et al., 2020). Nonetheless, parent‐child interactions continue to be implicated in the emotional and behavioral challenges of children with ID/DD (Fenning et al., 2014; Totsika et al., 2014). Although ABC has been adapted for other populations (Dozier & Bernard, 2019), this has not included children with ID/DD or those in lower‐resourced countries, which may potentially require special consideration.

Although parent training, more broadly, has been investigated for parents of children with developmental disabilities, with success (Hohfeld et al., 2018; McIntyre, 2008), the study of attachment‐based interventions, specifically, for this cohort has been limited (see, Mohamed & Mkabile, 2015; Platje et al., 2018; Sterkenburg et al., 2008). Weisz et al. (2013) also found reduced relative efficacy when youth interventions are applied outside of their original contexts. Thus, despite its promising evidence base, ABC's applicability in novel cultures and cohorts still needs to be established. To date, this has been investigated in studies exploring the experiences of first‐generation Latina mothers who received ABC (Aparicio et al., 2016), and that of intervenors (West et al., 2022) in the United States, as well as in European settings (Costello et al., 2021). The current study, in addition to the experiences of ABC recipients and intervenors, also explores the opinions of clinical experts regarding the implementation of ABC. Thus, the current study sought to explore the opinions, perceptions, and experiences of ABC from a range of stakeholders in order to inform the broader implementation of the intervention for children with ID/DD in South Africa and similar settings. This was achieved via the following objectives:

1. Exploring the opinions of experts on the strengths, areas for further development and recommendations for adaptations to the ABC for the South African context, and for families with a child with ID/DD.
2. Exploring caregivers’ perceptions of the ABC, or similar relationally‐focused, interventions for families with a child with ID/DD.
3. Exploring the experiences of the ABC of its first recipients and intervenors among South African families with a child with ID/DD.

## 2 METHODS

### 2.1 Design

An exploratory qualitative research design was utilized given the limited existing exploration into the opinions, perceptions and experiences of the ABC intervention, particularly in novel contexts and populations (Rendle et al., 2019). A two‐round Delphi procedure was nested within the broader qualitative design as a mechanism to gather insights on the ABC from clinical experts in the field of infants and young children with ID/DD (Brady, 2016; Fletcher & Marchildon, 2014).

### 2.2 Participants

Participants (*N* = 18) were sampled purposively and included: five experts with clinical expertise in working with children with ID/DD in South Africa, eight primary caregivers of children with ID, three primary caregivers of infants/toddlers with developmental delays who were recipients of ABC, and two trained intervenors who implemented ABC with these recipients (see, Table 1 for an overview).

**TABLE 1:** Profile of participants

Participant Group	Participant Description
*Experts* ^a^
Expert 1 (ST)	Speech‐Language Therapist. Over 10 years’ clinical experience with infants with developmental delays and disabilities.
Expert 2 (OT)	Occupational Therapist. Over 10 years’ clinical experience with infants with developmental delays and disabilities.
Expert 3 (ST)	Speech‐Language Therapist. Five years’ clinical experience with infants with developmental delays and disabilities.
Expert 4 (MT)	Music Therapist. Six years’ clinical experience with infants with developmental delays and disabilities.
Expert 5 (CP)	Clinical Psychologist. Over 10 years’ clinical experience with adults and children with intellectual disabilities and their caregivers.
*Caregivers* ^a^	
Caregiver 1	Biological mother (White, aged 51) to a son (14 years old) with non‐specific mild intellectual disability. At the time of the study, she was married and the full‐time caregiver to her children.
Caregiver 2	Biological mother (White, aged 50) to a son (14 years old) with mild intellectual disability associated with Down Syndrome. At the time of the study, she was married and employed on a part time‐basis.
Caregiver 3	Biological father (Black‐African, aged undisclosed) to a daughter (15 years old) with non‐specific mild intellectual disability. At the time of the study, he was divorced and remarried, and employed on a fulltime basis.
Caregiver 4	Biological mother (White, aged 47) to a daughter (13 years old) with mild intellectual disability associated with Down Syndrome. At the time of the study she was married and employed on a fulltime basis.
Caregiver 5	Maternal grandmother (Black‐African, aged 63) to a granddaughter (14 years old) with non‐specific mild intellectual disability. At the time of the study, she was widowed and employed on a fulltime basis. She had not formally adopted her granddaughter but was the beneficiary of a Foster Child Grant.
Caregiver 6	Biological mother (Black‐African, aged 45) to a daughter (2 years old) with Global Developmental Delay. At the time of the study, she was a single mother employed informally on a part‐time basis. She was a beneficiary of the Child Support Grant.
Caregiver 7	Biological mother (White, aged 38) to a son (10 years old) with mild intellectual disability associated with Down Syndrome. At the time of the study, she was married and employed on a full‐time basis.
Caregiver 8	Biological mother (Black‐African, aged 32) to a son (4 years old) with mild intellectual disability and Autism Spectrum Disorder. At the time of the study, she was a single mother employed on a fulltime basis.
*ABC Recipients* ^b^	
ABC Recipient 1	Biological mother (Black‐African, aged 39) of a child with developmental delays and cerebral palsy diagnosed shortly after birth. At the commencement of the ABC, the child's developmental age was estimated to be 14.2 months. The dyad completed all 10 sessions of the ABC over a period of four months.
ABC Recipient 2	Biological mother (Black‐African, aged 36) of a child with global developmental delay and epilepsy diagnosed within 6 months post‐partum. At the commencement of the ABC, the child's developmental age was estimated to be 5 months. The dyad completed all 10 sessions of the ABC over a period of four months.
ABC Recipient 3	Biological mother (White, aged 36) of a child with developmental delays diagnosed prior to age 2. At the commencement of the ABC, the child's developmental age was approximated to be 25 months. The dyad completed all 10 sessions of the ABC over a period of four months.
*ABC Intervenors* ^b^	
Coach 1	Clinical psychologist who, at the commencement of the ABC training, had five years of clinical experience. She trained as an ABC parent coach in 2019 and implemented the intervention with ABC Recipient 1, and attended weekly case supervision. Certification could not be pursued as a result of disruptions due to the COVID‐19 pandemic in early 2020.
Coach 2	Educational psychologist who, at the commencement of the ABC training, had three years of clinical experience. She trained as an ABC parent coach in 2019 and implemented the intervention with ABC Recipients 2 and 3, and attended weekly case supervision. She has subsequently received certification by the Attachment and Biobehavioral Catch‐up Laboratory (University of Delaware, USA).

*Note*: Within the ABC intervention protocol, intervenors are referred to as the “Parent Coach.” For the sake of brevity, they are referred as “coach” in this paper.

^a^ Information collected at the time of data collection.

^b^ Information collected at the commencement of the ABC.

Experts registered with the Health Professions Council of South Africa with at least 1 year's clinical experience working with children with ID/DD were eligible. An information leaflet was distributed to a network of health professionals via a gatekeeper. Interested professionals then contacted the principal investigator (PI) directly. The final expert panel consisted of two speech and language therapists (S.T.), an occupational therapist (O.T.), a music therapist (M.T.), and a clinical psychologist (C.P.).

Eight caregivers were recruited via primary schools for children with special educational needs and a non‐governmental organization (NGO) supporting families of children with Down Syndrome/ID. Adult primary caregivers of a child younger than 18 who had been diagnosed with an ID were eligible. An information leaflet was distributed to caregivers via teachers and the NGO administrator to invite participation.

The three ABC recipients and two intervenors (coaches) participated in a trial of the ABC in families of children with developmental delays (see Mohamed et al., 2022). These recipients had a child with ID/DD, had completed the ABC and were the only South African beneficiaries at the time of the study. The coaches received ABC training, had implemented it with the aforementioned recipients and were the only intervenors in South Africa at the time.

### 2.3 Ethical considerations

Ethical clearance was obtained from the Health Research Ethics Committee at North‐West University, South Africa (NWU‐00012‐18‐A1). Written and verbal informed consent was obtained from all participants prior to the commencement of data collection.

### 2.4 Data collection

#### 2.4.1 Experts

Individual semi‐structured interviews were conducted with each expert. Prior to the interview, experts were provided with a detailed written synopsis of the ABC. Participants were requested to familiarize themselves with the synopsis and prepare any comments for the interview. The interview schedule first covered the participants’ overall impressions of the ABC before probing more specifically for their opinions on the intervention's usefulness and strengths, concerns and areas for further development as well as recommendations for adaptations for children with ID/DD in South Africa. In a second round, the initial findings from the interviews were presented to the experts in writing. Participants were requested to review the findings, comment on their degree of endorsement of the findings, and provide any additional comments or input, in writing. A total of 60% of the experts participated in the second round and endorsed the interview findings with no disagreements or objections. Some additional comments were provided which were incorporated into the final analyses.

#### 2.4.2 Caregivers

Individual semi‐structured interviews were conducted with each caregiver in which they were asked to offer their perceptions of the ABC intervention, or similar interventions focused on supporting the parent‐child relationship, from their perspective as the primary caregiver of a child with ID. The interview schedule also explored the elements that facilitated their support for, and promotion of ABC.

#### 2.4.3 ABC recipients and intervenors

Individual semi‐structured interviews were conducted with each ABC recipient and coach within 1 month of having completed the intervention. Based on the participants’ first‐hand experiences of the ABC, the interviews—conducted by an independent research assistant—opened with a broad open‐ended question on how the participants experienced the intervention. The interview further explored experiences of the intervention process, of having used and received key intervention techniques as well as of the relationship between the parent and respective coach.

Interviews lasted on average 66 min, were conducted in English, and were audio‐recorded using a digital voice recorder. Recordings were subsequently transcribed verbatim. Prior to analysis, each interview transcript was checked against the audio file by the first author to ensure its accuracy. Furthermore, member checks were carried out with the caregivers, ABC recipients, and coaches to augment the validity of the findings (Candela, 2019).

### 2.5 Data analysis

Inductive thematic analysis (Braun & Clarke, 2006), using *Atlas.ti* (version 8 for Windows), was conducted by the first author and an independent Master's‐level research assistant. First, repeated readings of each interview transcript were carried out to become familiar with the overall contents. Thereafter, open coding was applied to the transcripts for significant segments with relevance to the aim of the study—this was done separately by the two coders who met to discuss the coding once all transcripts had been coded. Coding was then revised before preliminary themes and subthemes were developed separately by each coder. Themes were then discussed in a follow‐up meeting after which they were revised (merged, split, or discarded), where necessary, and finalized upon reaching consensus (see, Table 2 for an overview). This process was conducted separately for each participant group.

**TABLE 2:** Overview of themes and subthemes

Participant group	Theme	Subthemes
Experts	1. Intervention Strengths	Concordance with clinical interventions Intervention processes Role‐release function
	2. Points of Concern	Video‐recording Homework Intervention Timing
	3. Recommendations	Targeted psychoeducation and coach training Enhancing the family‐specific approach Engaging outside services Adapting in‐session activities Session flexibility
Caregivers (non‐ABC)	4. Focus on Caregiver‐child Relationship	
	5. Intervention Targets and Duration	
ABC Recipients	6. Benefits of ABC	Significant learnings Feedback: Homework Feedback: In‐the‐moment comments Feedback: Video‐feedback Working alliance
	7. Cultural Considerations	
ABC Intervenors	8. Focused and Targeted	
	9. Value of Feedback	Homework In‐the‐moment commenting Video‐feedback
	10. Supportive Supervision	Supervision process The supervisor
	11. Working Alliance	Trust, rapport and safety Caregiver commitment
	12. Challenges Experienced	Process of intervening Logistics

## 3 RESULTS

### 3.1 Experts

#### 3.1.1 Intervention strengths

**Concordance with clinical interventions**. For experts, the ABC synergized with the relational focus of their own discipline‐specific interventions. First, parallels were drawn between the ABC's practice of supporting caregivers’ identification of, and response to, children's signals and the facilitation, in speech‐language therapy, of communication as a dyadic process because “you can only have certain return interactions if somebody is giving back to you” (Expert 3). Second, experts’ use of approaches that help caregivers to identify and understand child‐cued communications converged with the ABC's emphasis on synchronous caregiver‐child interactions through following the child's lead. Expert 1, for example, noted her approach with caregivers to “observe, and then wait, and then listen [to] what the child is saying and respond in an appropriate way.”

Direct engagement with caregivers as agents of change in recognizing and attending to their child's signals was another noted point of convergence. For experts, this assisted caregivers of children with ID/DD directly to identify communications from the child that may be missed due to their idiosyncratic expression. This was also thought to provide an opportunity to draw caregivers—who may be withdrawn due to difficulties resolving the ID/DD diagnosis—more intensively into interactions with their child. Expert 2 noted that “it's hard to also interact with the child when you're not feeling that you're able to. So, I think the grieving plays a big role.” For Expert 5, the loss of the “normal” child could be “very traumatic [for caregivers]…that is really going affect attachments,” hence the active drawing in of the parent in the ABC was considered crucial.

**Intervention processes**. Home visiting and the strengths‐based coaching approach were highlighted as beneficial process‐related aspects. Home visitation was thought to enhance the convenience and reduce commuting time for caregivers of children with ID/DD who may be involved in multiple interventions. Home visiting was also believed to facilitate accessibility in remote areas so that the sessions could be done “next to the fire or the *kraal* [animal enclosure]…wherever the community or villagers reside” (Expert 5). This would allow access to an intervention that may otherwise have not been available to rural communities in South Africa. An added benefit of home visitation, according to the experts, was that this would allow for an immersion into the real world of the dyad in its daily routines, enhancing the relevance and generalizability of learnings.

In addition, the ABC's supportive, strengths‐based approach was highly regarded. Building on what the caregiver is already “getting right” was lauded by the experts because, in their experience, caregivers of children with ID/DD often feel ill‐equipped with regards to their caregiving. Hence, spotlighting and building on caregivers’ existing capabilities was regarded as crucially supportive of caregivers in challenging caregiving situations, contributing toward their degree of self‐confidence. According to Expert 4, “[caregivers] feel so dissociated from it all, hopeless, or they just want a bit of hope. So, I think, just to have that sense of ‘I've got this, even if I had it just today. Next week I'll try again’. Anything, so again that positive feedback, the confidence.”

**Role‐release function**. The ABC was also believed to provide a role‐release function. Experts felt that its contribution to capacity‐building allowed caregivers to rely less on trained professionals, lowering a heavy financial and time burden which is often highly proscriptive for many less privileged South African families. So, a brief intervention such as the ABC that builds caregivers’ capacities was regarded as attractive:

[What] I did like about this intervention and that it was with the primary caregiver that it, I felt you were going to do a lot of role‐release…especially in South Africa. Therapy is expensive. If you are able to role‐release…and make it practical. Wonderful! What a benefit to everyone (Expert 2).

#### 3.1.2 Points of concern

**Video‐recording**. Experts expressed concern around caregivers’ potential discomfort with being video‐recorded. It was proposed that caregivers of children with ID/DD who are already struggling may feel wary of having these difficulties with caregiving, and related insecurities, recorded: “…if a [caregiver] is already struggling to bond with their child…I don't know how comfortable they would feel with the video‐recording to start off with” (Expert 2).

**Homework**. Experts also raised concern about caregivers’ adherence to weekly homework tasks. It was felt that homework may be perceived as an added burden to the already overwhelming caregiving environment and busy schedule involved in caring for a child with ID/DD: “[caregivers] don't do homework. So, you know, you have a really difficult child, you have chores, you have all these things…almost a ‘Do you know what my day looks like?’. Now you're giving [them] something extra to do” (Expert 3). It was also noted that limited literacy in certain South African settings means that homework assignments involving reading and writing are additionally burdensome.

**Intervention timing**. Last, the proximity of the intervention to the diagnosis of ID/DD was raised as a possible concern. This was noted in relation to the possibility of caregivers being overwhelmed by the shock of the diagnosis, which may limit their degree of engagement with the ABC:

…at what point [is] this intervention implemented in the child's life?…If it's just after the diagnosis…the shock of the news that my child [has ID], how can I still provide nurturance to my child? (Expert 5).

#### 3.1.3 Recommendations

**Targeted psychoeducation and coach training**. Experts recommended integrating psychoeducation focused on developmental delays/disabilities into the intervention content. This was to increase caregivers’ awareness of alternative developmental trajectories to facilitate more realistic expectations of their child's competencies. For Expert 1, “I would add…developmental milestones so that you don't expect your one‐year‐old to communicate using words.”

Experts proposed that psychoeducation could also be used strategically to address perceived misalignments between the ABC and local culture. According to Expert 5, in many indigenous cultures in South Africa “elders or adults they like to be the ones to lead the way…and sort of learning the signals…from a child, might be tricky for them…but I do think that…our [caregivers] can be psychoeducated on the importance of that….” It was recommended that psychoeducation be used with cultural sensitivity, to promote intervention targets while taking into consideration localized cultural practices and understandings. Expert 5 advised, however, that these culture‐specific understandings first be engaged with intentionally to inform psychoeducation because “without giving room for the interacting or for listening…the psychoeducation can be completely blocked.”

Experts also recommended that coach training include specific instruction around ID/DD since this cohort may express their signals alternatively. Specific training in non‐verbal cues and communication, for example, was suggested could facilitate coaches’ ability to assist caregivers to identify, interpret and respond to their child's idiosyncratic signals, allowing them to “match” the child more readily and thus follow their lead. According to Expert 3:

…if you have no idea about child development, how are you going to be identifying the signals? And interpreting, ‘cause that's essentially what you have to do…I think [coaches] have to have knowledge in childhood development and how it is that you are going to interpret these cues.

**Enhancing the family‐specific approach**. Developing an experience‐near, ideographic understanding by engaging each family prior to the intervention was recommended to enhance the family‐specific nature of the ABC. It was believed this would further sensitize coaches toward cultural frameworks with which they may be unfamiliar but that may influence caregiving. Expert 4 suggested that observing,

how they just engage on a natural day is very key to how you're gonna inform [your intervention], because [no] matter who you are as a coach…your background and your bias and your information on what nurturance is [can interfere]…if you don't [understand] from their world what it means…

Therefore, developing an experience‐based understanding of each family could play a role in a culturally sensitive approach to ABC.

**Engaging outside services**. For the attachment relationship to be addressed adequately, experts believed that the dyad should be approached holistically. It was suggested that coaches receive training in when and how to activate referral networks, allowing caregivers to access adjunct support services when extraneous issues risk interfering with the ABC process. Expert 1 felt that “a possible way is to have connections with doctors…or with other health professionals…[which is] an infrastructure that can support them.” The experts also noted the role of social issues impacting dyads and interfering with intervention processes in South Africa, necessitating that the referral for social assistance be included in holistic case management. In addition to addressing broader psychosocial well‐being, this was also thought to prevent the misappropriation of ABC sessions by caregivers to address other issues, detracting from its focus. Expert 5 noted that caregivers are:

…overwhelmed and they always want to vent, so any sort of like attention given to them to talk about their difficulties, or how to manage their difficulties, it's always a space used for themselves, sort of like that…I would advise…to consider that.

**Adapting in‐session activities**. Experts recommended that in‐session activities for children with ID/DD account for their neurodevelopmental limitations. Expert 2 pointed out that “should you give an activity that's too hard they're going to get frustrated and they're not going to want to participate in that interaction….” She continued, explaining that children with developmental challenges may also have some degree of physical limitation (e.g., cerebral palsy) which may limit the kinds of activities they are able to participate in successfully. It was, therefore, recommended that tasks be tailored according to the individual child's capabilities so that they can benefit fully from the sessions.

**Session flexibility**. While the intervention content was considered applicable, experts recommended flexibility in how this content was organized such as when a dyad may be having difficulty with a particular theme and may require more intensive input on this. Experts suggested that additional sessions be considered to provide focused additional input when needed, for specific families to further support and augment changes in parenting:

I suppose it all depends on the families…looking at each family and seeing in each child and seeing well which session is going to need more than an hour…an extra session on each topic or whether they are okay with an hour…Being flexible (Expert 2).

### 3.2 Caregivers

#### 3.2.1 Focus on caregiver‐child relationship

Caregivers supported the ABC because of its focus on scaffolding the caregiver‐child relationship. As caregivers of children with ID, they felt this was particularly necessary because, in their perception, this relationship may be negatively affected by the diagnosis. It was pointed out that the caregiver's struggle—in their loss of the ‘normal’ child—to accept the diagnosis may interfere with the quality of the caregiver‐child relationship:

And I was getting very frustrated with it. So, and very often, I feel that's why he's leaning towards his dad. He loves his dad so much because he can feel, you know, I love him as much as I do, and nobody will know how much I do love him. But I think there's still a sort of barrier that's preventing me from really loving him (Caregiver 3).

Caregivers believed that being overwhelmed and emotionally less available to their child negatively impacted the quality of the relationship, affecting the child's feelings of safety. Additionally, the nature of caring for a child with ID may be experienced by the caregiver as a burden or punishment, further negatively affecting the caregiver‐child relationship, thus necessitating intervention:

These kids [with ID] need that relationship and a lot of parents probably struggle to relate to their own kids. Maybe because they're taking too long to understand them or it feels like a burden for them but I feel you know the bond must be so strong that it doesn't feel like a burden, it doesn't feel like a punishment. Even though you accept it you must just remember that your child depends on your well‐being as well in order for them to be who they are and be happy with that. So, it is an important bond to help strengthen (Caregiver 8).

Salient for caregivers was an acknowledgment of the importance of, and need for, relational support. This was predicated on the belief that the nature of the caregiver‐child relationship provides safety through the caregiver's sensitive responsiveness to the child. Hence, opportunities to support and strengthen this relationship, such as with the ABC, were regarded as necessary.

Participants felt the ABC facilitated the caregiver‐child relationship through supporting caregivers of children with ID. The active acknowledgment of their unique caregiving challenges was thought to build connectedness, counteracting the alienating effects of social stigma, thus supporting caregiver wellbeing. One caregiver felt that if she had taken part in the ABC when she first had her child, she “would have felt normal. I would have felt nurtured. I would have felt looked after. I would have felt there's somebody that cares enough to help me find my road” (Caregiver 1). Thus, the ABC, by working closely with the caregiver, was felt could play an important role in empowering them and expanding their support network.

#### 3.2.2 Intervention targets and duration

Caregivers perceived the clear delineation of intervention targets as useful because this facilitated identifying specific elements of caregiving that required attention, or were working well, making the learnings more actionable:

…it kind of made me go I could probably be able to identify some trigger point or some points that aren't working or some points that are working…[so that] I can quickly see where I am melting down which isn't working and be able to move out of it to make this interaction with [my daughter] a little bit better… (Caregiver 4).

Furthermore, caregivers felt that the 10‐session duration was intensive enough to impact caregiving with the support and guidance of the coach. It was noted, however, that this may depend on individual families’ needs and circumstances: “If that parent coach is the only person that mother will see during the week…she might want fifty sessions. Whereas a mother who's more independent in having nurturing from other places…then 10 sessions might be exhausting” (Caregiver 1).

### 3.3 ABC recipients

#### 3.3.1 Benefits of ABC

**Significant learnings**. Generally, the intervention appears to have catalyzed, for ABC recipients, improvements in how they understood and communicated with their children. For caregivers who had experienced early difficulties understanding their child's needs and for whom the anxiety involved in having a child with ID/DD distracted from getting to know their child beyond the diagnosis, these changes were experienced as particularly significant. According to Recipient 1,

If more mothers can get this especially the ones who get premature babies, it helps a lot…because of the stress you get stressed and sometimes…you can't relate to the kid and everything because of you're worried about [them] being sick and everything, so it helps you to be more open and…to be more observant to your child.

Similarly, Recipient 2 felt that the intervention was well‐timed because it came at a point when she was particularly overwhelmed which interfered with her ability to communicate with her daughter. So, being part of the ABC “gave me the tools to…communicate better with her…[and] interact better with my little one” (Recipient 2).

Specifically, the recipients highlighted learning to follow the lead as particularly salient for them as caregivers of children with ID/DD. Recipient 3, prior to the ABC, would “constantly try to teach my child something while she was playing,” leading most interactions. So, learning to take a step back, observe, and allow their children to lead was a significant learning, especially noting the impact this had on child behavior, such as increased confidence to explore their surroundings.

Recipients also highlighted learning to identifying voices from the past in relation to both nurturance and their use of frightening caregiving behaviors. One recipient felt that she had learned to listen to her own voice more after coming to realize that voices—from the past—had been influencing how she engaged with providing nurturance to her daughter, which taught her how to respond more sensitively. Recipient 3, similarly, reflected on her tendency towards frightening behaviors and how this had been ingrained in her caregiving due to having been parented in this way herself:

[Coach 2] said to me…as a parent, you can't be scary. So, you can't go round shouting at your child saying ‘YOU WILL NOT DO THAT!’…And that has been…the hardest habit that I've had to change, because my mother screamed at me for everything.

**Feedback: Homework**. Homework provided an opportunity for recipients to reflect on intervention content in relation to their own caregiving, which was consolidated when the homework was reviewed with the coach weekly, “[making] you re‐evaluate a situation” (Recipient 3). Although it was sometimes difficult to fit the homework into already busy daily schedules, it was found by the recipients to be beneficial. Homework served to reinforce recipients’ abilities to observe and attune to their child by providing a structure within which to do so which, for Recipient 2, made her “much more aware” of her daughter and her relational competencies and, for Recipient 3, served as a way of monitoring improvements over time which “re‐establishes that new habit that you're trying to learn.”

**Feedback: In‐the‐moment comments**. In‐the‐moment commenting from coaches was experienced as particularly useful by the recipients because it allowed them to develop insights into, and refine, their caregiving behaviors as they occurred. It helped Recipient 1 “to observe those things by [the coach] observing us and telling me ‘oh, this is good…doing this is good.’” It also provided encouragement and social reinforcement of target caregiving behaviors that were already in place through “spotlighting good,” which Recipient 2 felt “gave me the confidence that I'm doing the right thing.”

**Feedback: Video‐feedback**. Recipients reflected on the reinforcing quality of the video‐feedback which was experienced as positive, resulting in recipients learning more about themselves and their strengths and capabilities in relation to caregiving: “…sometimes I didn't even know that I'm doing that okay, that means okay that at least I'm doing good so I at least I've learned more about myself…” (Recipient 1). Recipient 2 felt that seeing herself on video was a critical aspect of her learning: “…sometimes you need somebody [to] tell you but when you see yourself doing it you realise, oh actually I'm not so bad. Maybe I'm just beating myself up unnecessarily.”

**Working alliance**. Reflecting on the relationship with their coach, recipients highlighted as significant the coach's attitude and demeanor as well as their support and guidance throughout the process. Across recipients, coaches were experienced positively, with reference made to coaches’ friendliness, warmth, professionalism, and understanding as characteristics that made recipients feel comfortable. The coaches’ non‐judgmental approach and attitude was noted for its role in softening more critically‐inflected feedback related to areas of development identified in sessions. All recipients experienced feeling supported and being guided by their coach in a collaborative partnership, in the absence of a top‐down approach. The warmth and support were experienced as holding during challenging moments and feelings of insecurity regarding caregiving competency, as well as during feedback, which normalized caregiving challenges and associated feelings.

#### 3.3.2 Cultural considerations

The recipients were all in agreement that the ABC and its foci were relevant to the South African context. They did, however, raise some thoughts regarding culture and caregiving that they believed were worth considering for the ABC in South Africa. Recipients reflected on learning, through their own childhood experiences, that children are to be controlled and managed by adults, or are to be ‘seen and not heard.’ This stands in contrast to the ABC's notion of following the lead and discourages considering, and taking seriously, children's feelings, stifling the provision of nurturance, another core ABC target:

…so for me [when I grew up] a child is a child. You just reprimand…you don't observe like maybe okay how does a child feel and how…is the child hurt or something and if the child is hurt, when I grew up it's like it's fine because you were naughty that's why… (Recipient 1).

Thus, there may be ‘culture shock’ for some families who subscribe to more traditional beliefs that allowing the child to lead or responding to them immediately when they are distressed, will ‘spoil’ them. Based on their experiences of the ABC, all recipients were of the belief that the intervention was applicable, culturally, and for children with ID/DD. They felt that buy‐in could be facilitated because “a person who is open‐minded would…receive [the ABC] well” (Recipient 2). The framing of the intervention upon introduction to potential beneficiaries—as an approach geared toward enhancing the caregiver‐child relationship rather than as something to rectify poor parenting—was felt could assist in obtaining buy‐in despite cultural differences.

### 3.4 ABC intervenors

#### 3.4.1 Focused and targeted

For coaches, the ABC's directive and specific nature that targeted clearly defined components facilitated the intervention's focus. In Coach 1′s experience, “[this] ensures that the targets get met and you don't get lost in…trying to reach all sorts of other therapeutic goals”. Both coaches also commented on how the home‐based format of the sessions supported the focused nature of the ABC because the sessions were based on in‐vivo interactions as they happened in the natural environments of the families involved, facilitating the applicability and transferability of intervention content. According to Coach 2, “doing it in the home is magic” because “you're right there in the middle of it, clothing lying around…kids taking you upstairs…And so, very quickly it becomes real, it becomes, well, you know what, this is us, deal with it.”

#### 3.4.2 Value of feedback

**Homework**. Flexibility around the completion of written homework was regarded as crucial for caregivers who are already overwhelmed and may experience this as additionally burdensome. Coach 2 believed that “the homework is important. But… I think the way that it's being sold, where it's not homework that you have to sit and do, but that it really is food for thought. I think it works well.” Thus, even when recipients were unable to complete the written component, reflecting with their coach on the task during sessions nonetheless allowed them to benefit from the homework.

**In‐the‐moment commenting**. Although both coaches found the delivery of in‐the‐moment comments initially challenging, they agreed that these comments were beneficial for recipients and impacted caregiving behaviors, which was encouraging. The coaches believed that the intensive, repetitive nature of the commenting delivered during naturalistic interactions in the home all, together, worked well to convey and cement the message, contributing toward what, and how, recipients learned:

… I think that repetitive nature helps it set in for the mom because it's kind of practiced in the moment. Then I also think the fact that it's happening within the moment while play is happening, that is also useful because it becomes less teachy… (Coach 1).

**Video‐feedback**. Video‐feedback was thought to augment in‐the‐moment commenting because recipients were able to concretely observe themselves in moments when they were doing well. Coaches felt that this was reinforced for the recipients who were able to witness their own progress over time. This made recipients increasingly aware of their evolving caregiving capabilities in relation to their child with ID/DD:

…it was very powerful to play moments back to the mom where she was doing more of the target or desirable behaviours that we were trying to work towards, and it actually ended up being more of an encouraging type of experience than I think making mom feel intimidated (Coach 1).

The supportive and encouraging nature of the feedback was foregrounded and valued by the coaches who themselves felt encouraged in the process: “I was genuinely excited with mom when she was getting it. So, there were many moments we were celebrating together” (Coach 1). Coaches also noted that video‐feedback may be especially valuable for caregivers of children with ID/DD in pointing out tendencies to be more “teachy” and lead interactions in attempts to stimulate development.

#### 3.4.3 Supportive supervision

**Supervision process**. Supervision was experienced as indispensable for its guidance on the application of in‐the‐moment commenting, which was new to both coaches. This was especially useful when working with dyads in which the child had ID/DD because it helped to reframe in‐the‐moment comments to be more appropriate given the child's functional limitations:

[The supervisor] was wonderful in that in saying, look, you're going to alter it slightly so you are going to say that this is going to help her feel safe, this is going to help her feel secure, this is going to let her know that you are there for her…This is going to help her regulate. So, the ones that you feel uncomfortable with, we'll wait with for a while…But by the end of it I was saying to her, well done mom, this is how you're building her vocabulary because I felt that comfortable that the programme was actually doing it (Coach 2).

Both coaches commented on the value of watching themselves on video during supervision, which mirrored the video‐feedback used in intervention sessions. Supervision played a crucial role in helping to fine tune observations to sometimes smaller and more subtle moments that may otherwise have been missed. Coach 2 elaborated on this further saying that “even the way [the supervisor] does supervision with us, she does in‐the‐moment commenting with us sometimes…And I'm like, ‘That feels good, do it more,’” mirroring the encouraging and supportive inflection of intervention sessions. So, much like the ABC sessions were experienced by the recipients, supervision was experienced by the coaches as supportive and non‐critical.

**The supervisor**. The abovementioned support was heavily facilitated by the nature of the supervisor who was experienced as containing, understanding, consistent, and reliable. This offered the coaches an experience of being held, which was especially beneficial in the context of families with a child with ID/DD where implementation of the ABC may be more challenging: “… there was an element of not just supervision but almost like a holding space when it got tough” (Coach 2).

#### 3.4.4 Working alliance

**Trust, rapport and safety**. Trust and rapport were experienced by coaches as particularly significant to their relationships with recipients. The safety of the working alliance was thought by coaches to have buffered against recipients’ uncertainties, mistakes, and insecurities, and was augmented by the ABC's strengths‐based, encouraging approach: “…it helps mom to feel like it's not her fault…and I think that was a good way to establish a therapeutic relationship” (Coach 1). This built trust and rapport which contributed to recipients feeling supported and joined in the process.

**Caregiver commitment**. Coaches also noted recipient openness and commitment to the intervention process as a key factor in facilitating the working alliance. Coach 1 noted how Recipient 1 “was incredibly willing to learn…and a very, very dedicated mother which made my job easier,” and for Coach 2, “…having them, even when they are questioning you and going ‘where's the science? This doesn't make sense.’ At least they're participating, they want to learn, they want to understand.” It was believed that this kind of active engagement and commitment was facilitated by recipients feeling like a collaborative partner in the process, allowing them to act as the agent of change and expert on their own children. Hence, the working alliance was strengthened by the collaborative nature of the partnership that characterized it, allowing recipients to feel like active agents.

#### 3.4.5 Challenges experienced

**Process of intervening**. Due to the coaches’ professional training as psychologists, the ABC was a paradigm shift for them. This was challenging because it involved bypassing their prior training and experience. They highlighted that boundaries had to be conceptualized differently to the psychotherapeutic approaches in which they had been trained. This manifested in the home‐visiting aspect of the ABC, which is not commonly a feature of traditional psychotherapies: “…the fact that it's taking place in someone else's home, I think this is very different in the South African context, we don't really go to people's homes to do therapy there. So, for myself, that was an adjustment as well” (Coach 1). Another notable challenge for the coaches was in‐the‐moment commenting. Not only was this highly directive, rather than exploratory, but it also involved actively and constantly interrupting the delivery of content to make well‐timed in‐the‐moment comments. Both coaches found this difficult to enact in the beginning, although this eased over time. Coach 1 reflected that,

when the mom is engaging in a target related behaviour you need to shift your focus and comment on that. So, you feel like you're interrupting yourself a lot. So, I found it was difficult at times to come back to the content again…it's just something to get used to.

The child's level of functioning was also a factor that complicated the delivery of in‐the‐moment commenting. Coach 2 found that the child's functioning affected interactions with their caregiver, limiting—earlier on—the coach's perception of the availability of relational material to comment on. Hence, implementing in‐the‐moment commenting was less straightforward. However, with the support of supervision, rethinking how the comments were delivered helped to manage this challenge. Supervision assisted in the process of lowering the threshold of which behaviors received in‐the‐moment comments such as, for instance, when “mom [was] responding to very slight, small cues…I had to be far more observant and aware…I had to find the things, so it was very hard for me” (Coach 2).

Another challenge raised was the potential for dissonance between the ABC and other therapies that families were accessing (e.g., physiotherapy). For Coach 1, such therapies encouraged caregivers to initiate and lead activities, contrasting with ABC's principle of following the child's lead. For children with ID/DD who are often involved in additional therapies to support different aspects of their development, this may require careful consideration when implementing the ABC. After noticing that Recipient 1 had started increasingly initiating activity, Coach 1 raised this with her and,

realised that this was [an] instruction that was given from physiotherapy…So my recommendation [would be] to take like a whole multidisciplinary approach if you are going to work with children that have developmental delays and incorporate [the ABC], so that there could be certain times for specific types of interventions. So, the mom can have a specific time where she does the physiotherapy where she would then initiate the tasks and then…for the majority of the time you want to create an interaction style where the mom follows the child's lead.

**Logistics**. The logistics—including time commitment—involved in being a coach were also challenging. This included traveling to and from families’ homes as well as ancillary activities related to supervision. Commuting to and from families’ homes was raised as time‐consuming, particularly where families lived significant distances away. It was also pointed out that although the families with whom the coaches worked lived in easily accessible and safe communities, many South African families do not. So, issues of physical access and safety in rural, peri‐urban, and township areas must be borne in mind for future implementation in South Africa: “So, you're kind of wondering where are you going to and will the area be safe for me to go to” (Coach 1).

While supervision was planned, the preparation for supervision added to the time burden and logistical challenges. Although highly valued, the process of self‐coding video segments to monitor fidelity was time‐intensive over and above the 90 minutes of supervision. This was complicated by weak internet connectivity which made uploading video‐recordings for supervision a laborious process: “…and it was coding, and it was video recording and it was cutting and uploading. Uploading [was the]…number one hassle…that would take sometimes the whole day” (Coach 2). Another logistical challenge was the time difference between Delaware and South Africa, which meant that the scheduling of supervision—at 6 p.m. or 7 p.m.—was not always convenient for the coaches.

## 4 DISCUSSION

Findings indicated positive perceptions and experiences of the ABC across participants, who supported the intervention and its implementation for children with ID/DD in South Africa. Framed as strengths, several factors contributed toward this support. The ABC was thought to coalesce with some other therapies because of its focus on the caregiver‐child relationship, which was regarded as key to the success of early intervention. With the caregiver‐child relationship as the ABC's inflection point, dovetailing with other therapeutic interventions could serve to facilitate a transdisciplinary approach in assisting families of young children with ID/DD. A transdisciplinary approach has been purported as the best practice for early intervention and could, therefore, advance the ABC as a component of an early intervention package to maximize development in children with delays (Guralnick, 2001, 2017). Role release—noted as a strength of the ABC—is regarded as contributing toward transdisciplinarity because this involves the integration of the target families into the team as collaborators (King et al., 2009). Partnering with, and capacitating, caregivers through early intervention during the first 1000 days of their child's life is also preventive, improves maternal mental health, and reduces the longer‐term reliance on professional services, which is beneficial in lower‐resourced settings (English et al., 2017; Tomlinson et al., 2022).

This was further highlighted in this study by coaches who were encouraged and motivated by recipients’ commitment to the process as collaborative partners rather than as passive observers. This active commitment also contributed to augmenting the working alliance, which was experienced positively by recipients who appreciated the support and warmth conveyed by coaches, feeling as though this support and encouragement contributed to their feelings of competence as caregivers. This is consistent with findings that show that positive interactions between therapist and client improve both the therapeutic alliance and treatment outcomes (Duppong Hurley et al., 2017).

The caregiver‐child relationship was regarded, across participant groups, as central to child development and wellbeing. The focus of the ABC on this relationship, therefore, reinforces its perceived relevance, generally, and for caregivers of children with ID/DD, specifically. These caregivers may feel less competent in their caregiving and lacking in caregiving self‐efficacy (Spiker et al., 2002). This may be rooted in the caregiver's difficulty identifying or understanding their child's needs during infancy when caregivers have not yet adapted to the potentially anomalous signal repertoire of their child with ID/DD (Vandesande et al., 2019b). By providing concerted and directed opportunities to facilitate—with supportive coaching—the caregiver's capabilities, the ABC may work to scaffold and shape their caregiving competencies. This is reified by the strengths‐based approach of the ABC in which caregivers’ already‐existing capacities are reinforced, which was a feature also highlighted by the mothers in Aparicio et al.’s (2016) study, and mentioned across participant groups in the current study.

Reinforcing caregiving capacities is anchored through the ABC's feedback system involving homework, in‐the‐moment commenting and video‐feedback. In alignment with the mothers in Aparicio et al.’s (2016) study, caregivers may be uncomfortable with being video‐recorded, especially parents who may be struggling. However, despite the initial discomfort, Aparicio et al. (2016) reported that the mothers became quickly accustomed to the video camera. The ABC recipients in the current study, however, did not experience any particular trepidation around being video‐recorded which may have been influenced by the benefits experienced in relation to video‐feedback such as observing themselves in the context of supportive feedback. This is in line with Balldin et al.’s (2018) conclusion that the use of video material can be an aid in interventions focusing on caregiver‐child interaction.

While video‐feedback is a notable aspect of the ABC, in‐the‐moment commenting is considered its ‘active ingredient.’ Although challenging to implement because it differs from commonly practiced exploratory approaches, its clinical value counterbalanced any initial challenges perceived by coaches in this study. This value was reflected by the ABC recipients who found these comments to be useful in understanding their children's needs and learning how to interact with them differently. These experiences correspond with the emphasis of in‐the‐moment commenting as the ABC's active ingredient which has been found to predict changes in parenting behaviors such as increases in parental sensitivity (Caron et al., 2018). In addition, the strengths‐based and supportive nature of these comments delivered in the context of a safe and containing working alliance may have further contributed toward the learnings experienced by the ABC recipients in this study (Aparicio et al., 2016).

Based on professional experiences of caregiver non‐adherence with homework, this component of the feedback system was flagged as a point of concern by experts. This is consistent with others who have found homework to be a difficult component of parenting programs for caregivers to follow (Christian et al., 2014), and that despite its benefits, adherence to homework is suboptimal (Danko et al., 2016). A regularly reported reason for poor homework adherence is a lack of time (Chacko et al., 2013). However, while the ABC recipients did not always complete written tasks, this was not experienced as a barrier in sessions. This is attributed to the fact that the ABC process is not reliant on the written homework and that even in the absence thereof, the skills are reinforced and reflected upon in person. Hence, the engagement with the caregiver is always retained even when the actual written task has not been completed. For coaches, the value of the homework was situated in the reflective engagement with the caregiver rather than the written document. This is consistent with Clarke et al.’s (2015) findings that it is the extent to which caregivers are actively engaged in the process that is most important in predicting intervention response. In this study, the flexibility around the homework appeared to ‘free up’ caregivers by removing an additional obligation while not sacrificing caregiver engagement, which was further augmented and held by the safety of the working alliance. As an added benefit, the non‐reliance of ABC on reading or writing in relation to homework circumvents literacy inequities in South African communities (Khumalo & Alhassan, 2021).

The issue of caregiver emotional withdrawal in response to the child's diagnosis of ID/DD was raised in the context of the expert and caregiver interviews. Grief and sadness are common caregiver responses to the diagnosis of ID/DD (Van der Weck et al., 2021), and may be persistent longitudinally (Fernandez‐Avalos et al., 2021) although, with time, many are able to develop a new perspective characterized by possibilities (Van der Weck et al., 2021). Kielb et al. (2019) reported that depression and posttraumatic stress may be particularly acute early on due to the shock associated with the diagnosis. The timing of the ABC is, therefore, important to consider and was raised as a point of concern by the experts. Concurrently, this also highlighted the significance of the mental and emotional health of caregivers of children with ID/DD and the impact of this on attachment (Alexander et al., 2018). This underscores, again, the importance of the relational focus of the ABC for this cohort where parental emotional withdrawal may be more prevalent due to the lack of resolution, or non‐acceptance, of the child's diagnosis, affecting the degree of caregiver sensitivity. Although there was concern about the timing of the intervention in relation to the child's diagnosis, this was counterbalanced by the experiences of the ABC recipients who felt supported through the intervention under overwhelming and challenging circumstances. The active engagement of the caregiver by the ABC may have contributed toward them feeling supported by their coach—reflected in the positive experiences of the working alliance—and may have built their confidence and competence as they observed changes in their child through having their own capacities reinforced and encouraged. Therefore, rather than disengagement serving as a barrier, the ABC, through its actively engaging processes, supportive coach, strengths‐based approach, and consistent, multi‐pronged feedback, may have worked to counteract withdrawal during a period critical for the establishment of attachment.

The ABC—albeit indirectly—may have a role to play in counteracting the effects of stigma for parents and caregivers of children with ID/DD. Stigmatizing experiences in relation to their child's disability are commonly reported among caregivers in South Africa (Malatji & Ndebele, 2018; Mkabile & Swartz, 2021; Mmangaliso & Lupuwana, 2021; Modula, 2022) and other African contexts (Aldersey et al., 2018; Masulani‐Mwale et al., 2016; Oti‐Boadi, 2017; Tekola et al., 2020; Tilahun et al., 2016; Zuurmond et al., 2020). This may be particularly acute in township and rural communities where a combination of factors such as poor education, poverty, and unemployment may magnify stigmatizing experiences related to having a child with ID/DD (Rohwerder, 2018; Zuurmond et al., 2020). In addition, in African contexts, stigma is further fuelled by the culture‐bound meaning‐making of disability as resulting from demon possession, witchcraft or similar causes (Mkabile et al., 2021; Scior et al., 2020). These experiences of stigma may have an alienating effect on both the person with ID and their caregivers resulting in isolation from extended family and the wider community and leading to psychological distress (Ali et al., 2012).

In light of this it is important to note that the ABC‐program—in alignment with the social model of disability (Kattari et al., 2017)—takes a strengths‐based, supportive and encouraging approach in which coaches convey compassion and understanding, in contrast to the blame and ostracism that families may sometimes meet. Through the unconditional acceptance and acknowledgment of the parent by the coach as well as the facilitation of parents’ confidence and self‐efficacy, which may encourage them to seek out supportive connections that are similarly accepting, the ABC may contribute toward rebuilding social connectedness for these caregivers and families which may protect and promote their mental health (Kawachi & Berkman, 2001; Saeri et al., 2018). This points to the more interpersonal—as opposed to purely ‘professional’—role that coaches may play through creating opportunities for meaningful social connection which McConkey et al. (2016) refer to as a “professional friend” who contributes to a broader coalition of support for families that have a child with ID/DD (McKenzie & Chataika, 2018). Deitz et al. (2020) found, for example, that the social aspects of a home‐visiting community health program were considered equally as meaningful as the professional, disease management aspects, highlighting its significance. This suggests that the core targets—the clinical aspects—of the ABC as well as its supportive role—the interpersonal connectedness aspects—may be key assets for families of children with ID/DD, especially in communities where stigma, isolation and exclusion remain significant social issues such as in under‐resourced communities in South Africa (Mkabile & Swartz, 2021). While South Africa has, to a limited degree, achieved steps toward greater inclusiveness—through attempts at deinstitutionalization, for example—people with disabilities and their families remain isolated within their communities and rarely participate in community life (Capri et al., 2018). The ABC can, therefore, also—on the one hand—make some contribution toward greater inclusiveness by helping caregivers provide their child with ID/DD with a secure base from which to explore social interactions with other people, complementing efforts to make local communities more inclusive. On the other hand, it can also facilitate inclusiveness for the family in community life by expanding their network of support.

### 4.1 Practice and training recommendations

**FIGURE 1:**
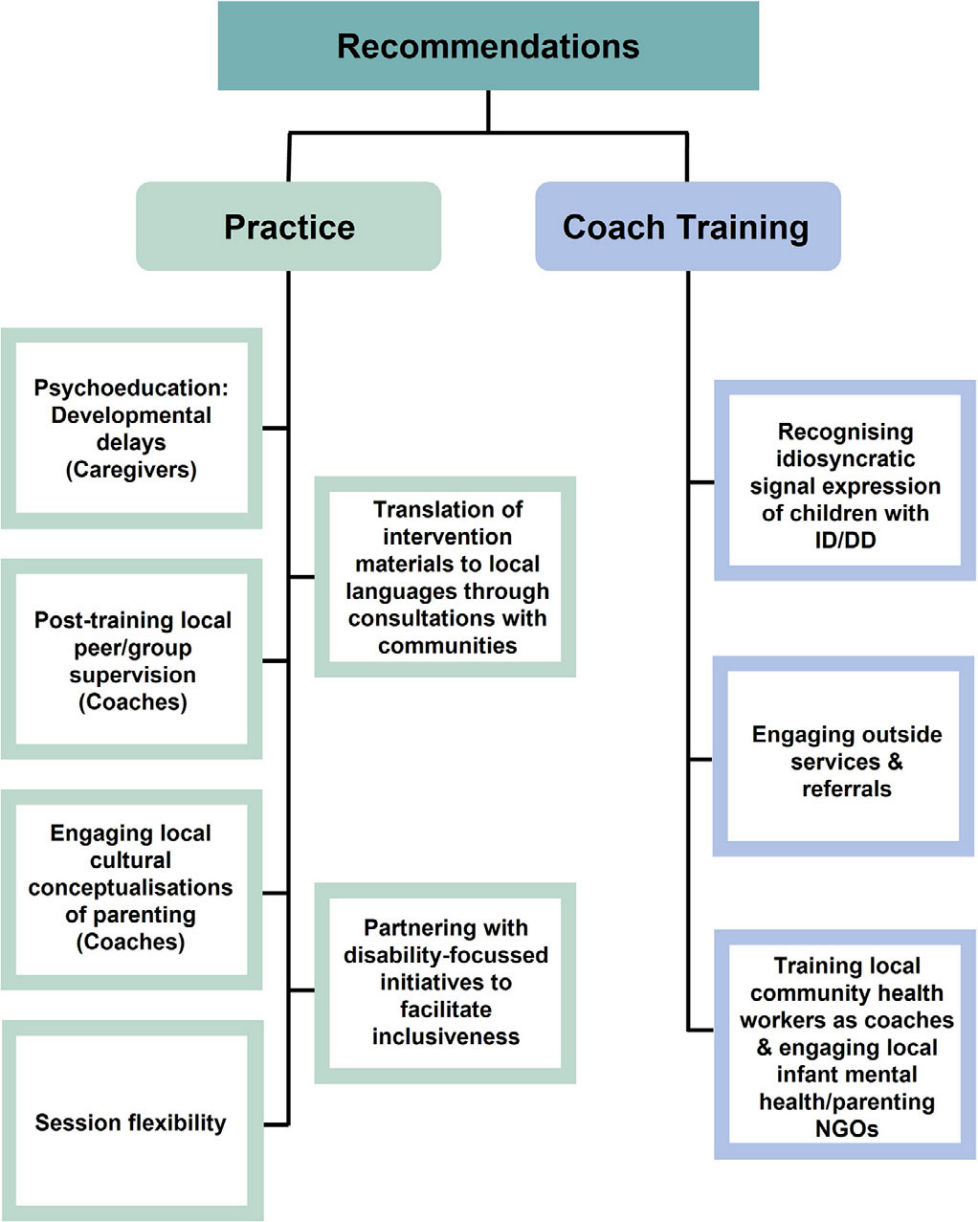
Practice and training recommendations

Based on this study's findings, some pertinent recommendations (see Figure 1) are offered for adapting certain ‘surface elements’ that would retain the ABC's core intervention identity while facilitating the implementation of the intervention for families that have a child with ID/DD in South Africa, and similar sociocultural contexts (Barrera & Castro, 2006).

In the context of working with children with ID/DD, accurate knowledge—through targeted psychoeducation—around delayed development may facilitate more realistic expectations from caregivers of their children's competencies. This allows for improved understanding and caregiving responses that are better aligned to the child's signals and behaviors given their functional limitations. Furthermore, adjusting the threshold for the behaviors that warrant in‐the‐moment commenting may be necessary to avoid coaches misidentifying, or missing, moments of significance due to the subtle and idiosyncratic expression of signals by children with ID/DD (Vandesande et al., 2019b). Therefore, the integration of knowledge on attachment and ID/DD into coach training is also essential. It may also be necessary to adjust in‐session activities to align more appropriately with the functional capacities of the child. This also underscores the importance of supervision in shepherding coaches in adapting in‐session activities and in identifying and responding to relevant behaviors that may be less clearly expressed when working with families who have a child with ID/DD.

The use of in‐the‐moment commenting and video‐feedback in supervision allowed for experiential learning that was perceived by coaches as making an invaluable contribution to the implementation of the ABC in families with a child with ID/DD. The supervisory relationship, not unlike the relationship between coach and caregiver, can serve to foster a transformative, confidence building quality in coaches (DeCandia Vitoria, 2021). This may be particularly necessary when implementing a new approach and in novel populations such as children with ID/DD where the application may be less straightforward. In addition to one‐on‐one supervision, peer and group supervision among local coaches may provide continual, and more widespread, support to coaches in the longer term.

Despite the perceived relevance of the intervention to South Africa, consideration of culture in its implementation is nonetheless recommended. Culturally‐informed beliefs about caregiving (e.g., children must be seen and not heard) may misalign with ABC targets, undermining intervention effectiveness (Super & Harkness, 2002). In addition, the manner in which individual families understand and enact target behaviors such as nurturance could be uniquely based on cultural practices (e.g., tying the infant to the mother's back), emphasizing the need to first engage and develop an understanding of each family so that the intervention is appropriately tailored (Van Horn et al., 2012). By engaging families on their beliefs about parenting, coaches should actively work toward the development of cultural competence, which has been identified as an important route to improving the outcomes and experiences of beneficiaries that differ in background to the intervenor (Ertl et al., 2019).

Although language was not raised specifically in this study, language and culture are inextricably linked (Jiang, 2000). While the ABC recipients in this study were fully conversant in English, this is not the case for a significant proportion of the South African population. Sattler et al. (2022) have noted challenges in this regard when implementing ABC among Spanish‐speaking families in the United States and suggested that intervention materials need to be translated because in‐vivo interpretation of English materials led to inevitable losses in translation. Translation of ABC materials has been successfully achieved in other international contexts in which the intervention has been implemented among rural communities through using words and phrases like those in English but reflective of linguistic differences between languages and making context‐specific video material with local ‘actors’ using the local language (Costello et al., 2021). Translation of intervention materials might be most effective if done in collaboration between ABC developers and local partners in South African communities so that linguistic and cultural issues can be considered through bidirectional knowledge‐sharing as well as capacity‐building (Costello et al., 2021).

In line with other research from developing contexts, the current study's findings suggest that the emotional health of parents of children with ID/DD (John, 2012) as well as their social realities (Masulani‐Mwale et al., 2018) must be accounted for because this could influence parents’ full engagement in, and benefit from, the intervention. This is consistent with Aparicio et al. (2016) whose participants suggested, similarly, that addressing basic needs was important when implementing the ABC among disadvantaged families. Coaches, therefore, require an awareness of psychosocial health. It is recommended that coaches receive training in caregiver mental health and social issues to facilitate an understanding of when to refer and how to access and activate appropriate referral networks. Thus, where the caregiver or infant experiences extraneous medical, psychological, or social distress that impacts on the intervention process and success, these can be addressed through referral and appropriate case management. Dozier et al. (2011) stress the importance of adhering to the protocol to avoid the intervention becoming ‘hijacked’ which, as noted in this study, may happen with vulnerable families where larger systemic stressors are at play. Thus, it is important to attend to securing referral networks so that these additional, albeit distal, stressors can be addressed without derailing the focus of the ABC.

While there are important points of convergence with other interventions, there may also be risk of conflict with ABC. Dozier and Bernard (2019) have noted that services such as physiotherapy, for example, may inadvertently discourage caregivers from following the child's lead by turning interactions into learning opportunities. For infants with ID/DD who are regularly beneficiaries of multiple simultaneous treatments (Heiman, 2002), it may be pertinent to consider how the ABC fits into the broader intervention landscape for individual families. Adjunct interventions are often necessary to stimulate developmental competencies for children with ID/DD. Hence, this recommendation is intended to encourage greater integration of services and multidisciplinary collaboration, allowing the ABC to harmonize with other interventions, limiting points of conflict.

It may also be beneficial—in addition to integration with clinical interventions—to partner ABC with existing disability initiatives to provide a holistic approach consistent with the social model of disability while still addressing, specifically, the parent‐child relationship. For instance, the partnership between Shonaquip and the Uhambo Foundation—Shonaquip Social Enterprises—in South Africa involves the provision of mobility devices to people with ID in conjunction with empowerment, educational and advocacy initiatives to influence policy as well as enhance access and inclusion for people with ID and their families (see Trafford et al., 2021). To flourish, children with ID/DD must be provided with various kinds of support that act synergistically to protect, uplift and empower (Trafford & Swartz, 2021). Thus, in addition to interventions such as policy development, assistive technologies, social assistance and the like, the ABC could serve as an additional branch in the broader system of available support to children with ID/DD and their families. Philpott and Muthukrishna (2019) also note that parents of children with ID/DD must be included as collaborators and active agents in these support endeavors. The ABC's approach aligns with this—as reflected by the ABC recipients in this study who experienced the process as a partnership—through its active drawing in of caregivers and capacitating, and empowering, them in their caregiving.

Although logistical issues emerged as a challenge for coaches, this could be minimized by training coaches from within local communities who could subsequently serve as community‐based supervisors. This would address issues around travelling time, safety, and access in township and rural communities. In addition, this could assist with navigating the cultural landscape by accessing the emic perspectives of these local coaches to benefit the integration of local cultural knowledges around caregiving, attachment and development with the existing foci of the intervention. South Africa may be well‐positioned to do this given its history of using Community Health Workers (CHWs) to improve access, in lower income and rural communities, to health services, in general (Murphy et al., 2021), as well as attachment‐based interventions, specifically (Cooper et al., 2009). Furthermore, NGOs in the fields of caregiving and infant mental health may be a valuable resource with well‐established relationships in local communities that could be utilized to facilitate contextually‐sensitive community implementation of the ABC. It is, therefore, encouraging that the ABC has retained its effectiveness when implemented in community settings (e.g., Roben et al., 2017). However, it is also acknowledged that the adaptation and implementation in South African community settings may come with its own unique challenges and that the apparatus for consultation as well as careful monitoring and evaluation will need to be put in place to further facilitate scaling up of the intervention, locally. Additionally, by partnering with disability‐focused initiatives (e.g., Chaeli Campaign) in training local CHWs as ABC coaches, further progress can be made toward promoting and encouraging disability inclusiveness within their communities, removing barriers for families of children with ID/DD to accessing support and services.

Last, the issue of the flexibility around sessions was raised in this study in relation to how caregivers may respond to the intervention, suggesting that additional—or fewer—sessions may be required depending on the strengths and weakness of individual families (Bernard et al., 2013). This is in line with the recognition by Dozier et al. (2018, p. 24) that “[adjusting] dosage according to parent response to treatment seems an important next step” in optimizing the ABC for individual families. This could be beneficial for families with a child with ID/DD where there may be unique caregiving experiences that require more, or less, intensive input.

### 4.2 Limitations

First, the study was limited to only five participants who had participated directly in the ABC as these were the only local stakeholders at the time of the study. Future larger group‐based studies should seek to explore the accounts of a wider variety of stakeholders across implementation sites to further extend knowledge on the use of the ABC in novel settings and populations rooted in first‐hand accounts, which may inform more considerable adaptations than what has been possible with the current sample. Second, having had positive experiences of the ABC may have introduced a selection effect in that the ABC recipients may also have chosen not to participate in this study had their experiences of the ABC been unfavorable. The study may, therefore, not be best positioned to comment on what makes the ABC especially appealing or unappealing to potential beneficiaries. Third, only one participant on the expert panel was of African ethnicity, limiting exploration of indigenous African cultural themes related to the ABC. Future research is encouraged to sample experts from a wider range of indigenous groups to broaden insights into the ABC's cultural applicability. Last, the ABC was also only implemented in families proficient in English which did not permit the exploration of the complexities and challenges of implementation in other local South African languages. Given that a significant proportion of the South African population, and those in lower‐resourced settings, may not be English‐proficient and may require the ABC in another of South Africa's 11 official languages to access its full benefit, future research should seek to explore language‐related adaptations that are specific to the South African context and its multilingual landscape.

## 5 CONCLUSION

This was the first study to explore the opinions, perceptions and experiences of the ABC for children with ID/DD in South Africa from experts, caregivers as well as ABC recipients and coaches. Overall, there was notable support for the ABC across participant groups who identified with the foci of the ABC and its approach to supporting the caregiver‐child relationship, which it was noted was needed for caregivers of children with ID/DD. The strengths‐based, supportive and encouraging coaching approach combined with repeated positive, reinforcing and directed feedback within the context of a trusting and holding working alliance was considered to be, and experienced as, a safe environment for caregivers of children with ID/DD who initially found understanding their children's needs challenging. This facilitated significant learnings for those who received the ABC primarily related to interacting with their children differently. Coaches were similarly supported through quality supervision and encouraged by caregiver commitment to the process. Although some concerns were raised and challenges were experienced, these reflections informed pointed recommendations which included, among others, training coaches in identifying anomalous communications and in activating referral networks, identifying and training local and community‐based coaches and partnering with NGOs to address logistical as well as context‐ and culture‐related factors involved in implementing the ABC in a novel setting.

## CONFLICT OF INTEREST

The authors declare that there are no potential conflicts of interest.

## Data Availability

The data that support the findings of this study are available on request from the corresponding author. The data are not publicly available due to privacy or ethical restrictions.
